# The best of both worlds: photosynthesis and Solanaceae biodiversity seeking a sustainable food and cosmetic industry

**DOI:** 10.3389/fpls.2024.1362814

**Published:** 2024-02-16

**Authors:** Cosette Aguirre-Bottger, Gaston Zolla

**Affiliations:** Grupo de Investigation en Fisiología Molecular de Plantas, Facultad de Agronomia, Universidad Nacional Agraria La Molina, Lima, Peru

**Keywords:** photosynthesis, Solanaceae, sustainability, biodiversity, cosmetics

## 1 Introduction

The global food supply crisis is one of humanity’s most significant risks (World Economic Forum, 2023). Climate change is causing the loss of natural resources, which is closely related to this crisis (Mirzabaev et al., 2023). Therefore, it is crucial to implement sustainable food systems that ensure food security for both present and future generations. Thus, food should be available, accessible, and nutritious (Peng and Berry, 2018). Delaying the implementation of these goals will contribute to food insecurity and lead to a more polarized world.

Improving photosynthetic efficiency is critical to ensure food security because it generates 90% of plant biomass (van Bel et al., 2003) and increases crop yield (Brestic et al., 2021). However, photosynthesis is affected by high temperatures (Mathur et al., 2014), irregular rains (León-Sánchez et al., 2016), and drought (Wang et al., 2018), among others. The persistence and severity of these phenomena reduce the photosynthetic rate, exerting selection pressure mainly in C3 plants (Sello et al., 2019), affecting their adaptation biodiversity and could lead to an irreversible loss of genetic diversity (Demír, 2021), which is relevant to implement sustainable food production systems through genetic improvement (Salgotra and Chauhan, 2023).

The Solanaceae family is a prime example of climate change vulnerability because their centers of origin are in countries highly vulnerable to climate change (Samuels, 2015). In this regard, Solanaceae is among the 12 most diverse plant families, and more than 1,500 native species can be found in South America alone, and Peru standing out for its diversity (Palchetti et al., 2020). This richness translates into genetic and metabolic diversity that can be useful to improve the crop photosynthetic rate. Therefore, it is essential to identify the critical genes for light and dark phases.

## 2 The underlying genetic architecture related to photosynthetic efficiency:

**Figure 1:**
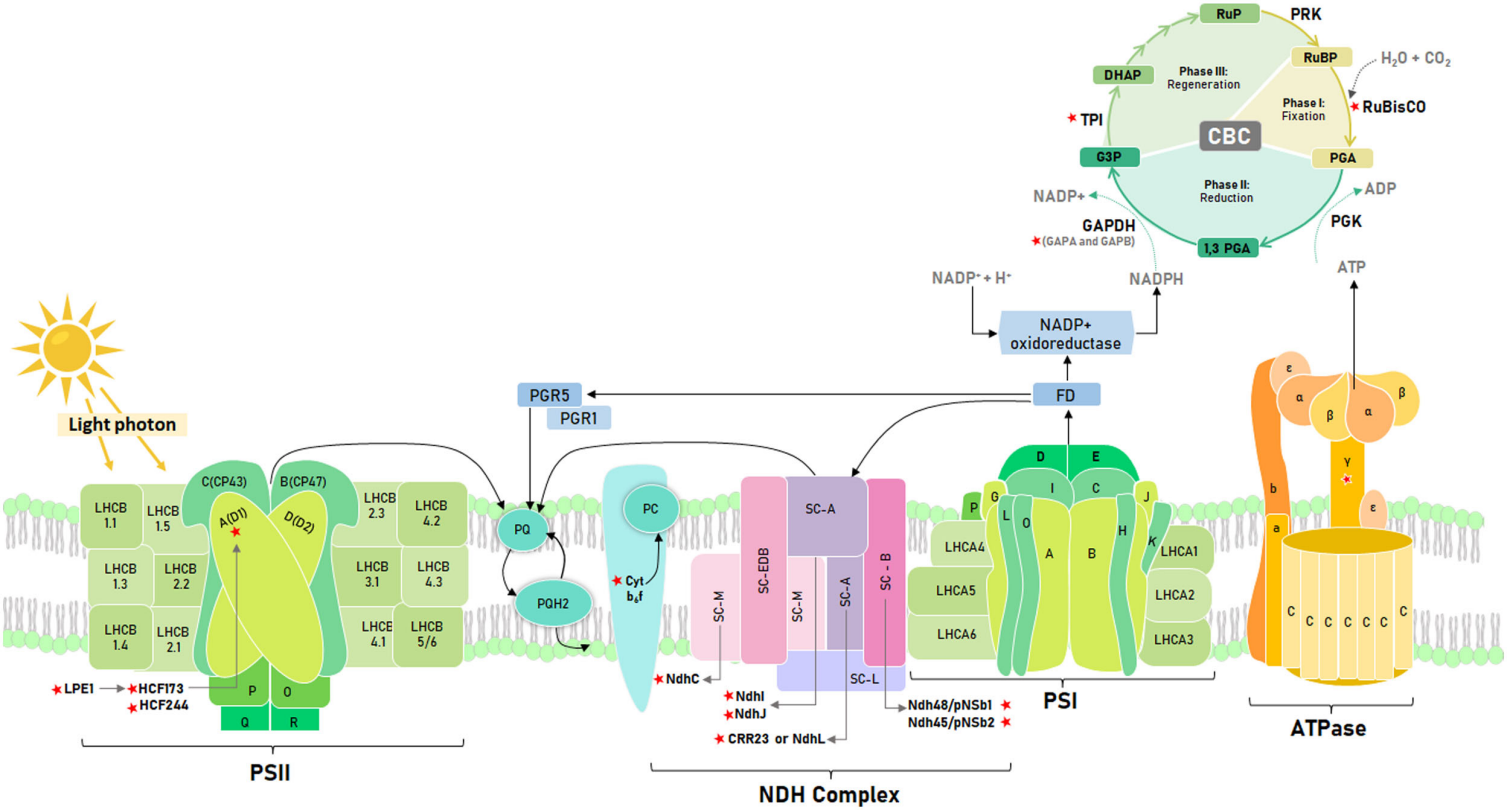
Schematic representation of the photosynthetic electron transport chain and the Calvin Benson cycle. Key photosynthetic genes identified with red stars. Light phase: Low photosynthetic effciency 1 (LPE1), High Chlorophyll fluorescence phenotype 173 (HCF173), High Chlorophyll fluorescence phenotype 244 (HCF244), D1 reaction center (D1), Cytochrome b6f complex (Cyt b_6_f), NAD(P)H dehydrogenase-like (NDH) complex subunits (NdhC, NdhI, NdhJ, NdhL or CRR23, Ndh48 and Ndh45), ATP sintase gamma subunit (γ Subunit). Dark phase: Ribulose bisphosphate carboxylase oxygenase (RuBisCO), Glyceraldehyde-3-phosphate dehydrogenase subunits (GAPA and GAPB) and Triosephosphate isomerase (TPI). Electron flow represented with black lines.

Regarding the light phase (**Figure 1**), Leister (2023) proposes a list of genes, including the D1 gene of the photosystem II reaction center (PSII), that improve photosynthetic performance and plant growth when overexpressed. Thus, Chen et al. (2020) showed that *Arabidopsis* transgenic lines that overexpress D1 doubled their biomass under thermal stress (42°C). The D1 biosynthesis is mediated by genes that ensure psbA correct translation (Zhang et al., 2000). Thus, LPE1 binds to the 5’ end of psbA to facilitate association with HCF173 (Jin et al., 2018), which prevents exonucleotide degradation of psbA mRNA, ensuring its binding to the ribosome (Bollenbach, 2003). In addition, HCF244 is co-expressed with HCF173, which encodes a gene necessary for the translational initiation of psbA and stabilization of this messenger RNA (Link et al., 2012). These genes are relevant for plant development, in *Arabidopsis lpe1-3* mutant showed a 70% reduction in the rosette size, and a drastic reduction in the ratio of variable fluorescence to maximum fluorescence (Fv/Fm) (Jin et al., 2018). For *hcf173*, Link et al. (2012) also had a similar reduction in rosette size in *Arabidopsis* than *lpe1-3*. RNA is highly unstable in *hcf173*, leading to a drastically impaired accumulation of PSII polypeptides (Schult et al., 2007). On the other hand, the *hcf244* mutant cannot grow under autotrophic conditions due to a drastically impaired accumulation of PSII proteins (CP47, CP43, D1, and D2); reaching only about 10% to 20% of wild-type levels (Link et al., 2012). Both, HCF173 and HCF244 were identified by Bhattacharya et al. (2023) in tomato stromal proteome as part of the 29 orthologous proteins involved in the assembly, stability and repair of the PSII complex.

Another critical component of the electron transport chain is the cytochrome b6/f complex (Rochaix, 2011). Mutants in tomatoes of the petM subunit of this complex showed lower electron transport rate, CO2 assimilation, and carotenoid content than the wild type. In addition, mutants of the petM-4 line showed late autotrophic growth (Bulut et al., 2023). In tobacco, petA, B, and D mutants showed a lower content of thylakoid membranes (Monde et al., 2000). Considering that the cytochrome b6f complex regulates the acclimation of photosynthetic organisms to changing light conditions (Malone et al., 2021) and that algae have an exceptional ability to adapt to such conditions (Sukenik et al., 1987), Yadav et al. (2020) identified that tobacco specimens transformed with cytochrome b6 gene from *Kappaphycus alvarezii* have a net photosynthetic rate higher than the wild type by approximately 60%. This improved performance was also evident in the growth and starch accumulation of the transgenic lines. Yadav et al. (2018) reported similar results with transforming tobacco specimens with the UfCytb6 gene from *Ulva fasciata*. These findings show that photosynthetic and growth enhancement of tobacco specimens through manipulation of cytochrome b6f subunits is a potential way to improve their performance in light-changing environments.

In addition to the structural genes of PSII, the NADPH dehydrogenase or NDH complex is also relevant for the light phase since it participates in the cyclic transport of electrons to maintain the balance of the redox system to mitigate oxidative stress in the photosynthetic apparatus (Ma et al., 2021). The *CRR23*, *NDH48*, and *NDH45* subunits guarantee the accumulation and stabilization of this complex in *Arabidopsis*. Thus, the *crr23* mutant showed a 12.5% reduction in the accumulation of the NDH complex (Shimizu et al., 2008), while *ndh48* and *ndh45* revealed functional deficiencies of this complex (Sirpiö et al., 2009). On the other hand, in tobacco, a 25% reduction in the photochemical efficiency of PSII was identified in mutants for the C, J, and K subunits due to the increase in ROS at -4°C and 42°C (Wang et al., 2006). It is crucial to study subunits that cause a decrease in photosynthetic efficiency because of temperature stress. Mutations in these subunits can negatively impact the plant’s ability to withstand frost. Therefore, it is essential to investigate these subunits in potato wild relatives as a potential solution to this problem (Nicolao et al., 2023).

On the other hand, ATPC1, the γ subunit of ATP synthase, induces conformational changes in the catalytic region of this enzyme that are necessary for ATP synthesis (Cheuk and Meier, 2021). It possesses two cysteine residues that regulate ATP synthase activity in response to fluctuating intracellular redox conditions due to the unstable activity of the photosynthetic electron transfer chain associated with changing light intensity (Akiyama et al., 2023). In *tobacco*, Rott et al. (2011) identified that atpc1 mutants showed a reduction of more than 50% in growth after 14 weeks and also a reduction in the chlorophyll a/b ratio; this change suggests a rearrangement of the photosynthetic apparatus. Furthermore (Kohzuma et al., 2013), identified that the knockout of *atpc1* in *Arabidopsis* cannot perform autotrophic growth.

On the other hand, overexpression of enzymes in the dark phase (**Figure 1**) does not necessarily result in improved photosynthetic efficiency. According to Zhao et al. (2021), balancing the catalytic activity of the different enzymes in the Calvin Benson Cycle (CBC) is crucial. RuBisCO is one of the most essential enzymes studied for enhancing photosynthetic efficiency, biomass accumulation, and crop yield (Lin et al., 2021). An evaluated strategy to make the catalytic activity of RuBisCO more efficient is to increase the concentration of CO_2_ around this enzyme through synthetic engineering, as this could increase the photosynthetic efficiency of C3 plants by 25% (Zhu et al., 2010). In nature, a greater availability of foliar CO_2_ was observed in *Solanum pennellii*, a wild relative of tomato, where the distribution of its stomata limits the diffusion of CO_2_ by photorespiration, facilitating its fixation and a consequent higher photosynthetic rate (Muir et al., 2014). However, despite the greater availability of CO_2_, a limiting factor is the catalytic inefficiency of RuBisCO compared to CO_2_ and O_2_ as substrates. The short subunit of RuBisCO controls the affinity regulation of these molecules (Genkov et al., 2010). In tobacco, mutations in this subunit have been found to reduce the total content of RuBisCO by 93% and biomass accumulation by 90% compared to the wild-type.

Despite the recent improvements, Lin et al. (2021) suggest that the most effective way to enhance the RuBisCO efficiency is to modify the long subunit; the active site of the enzyme is located there, making it vulnerable to changes in temperature and humidity. In this context, previous studies have identified that high temperatures and dry environments can reduce RuBisCO efficiency by up to 40% (Parto and Lartillot, 2018). This scenario is common for many crops, which may experience a loss of productivity ranging from 3 to 13% for each one-degree increase in temperature (Zhao et al., 2017). Indeed, Lin et al. (2022) conducted a study to address a problem related to RuBisCO efficiency in hot and dry environments. They explored the potential of thermostable RuBisCO ancestors in Solanaceae and found that they have superior catalytic efficiency, suggesting that by utilizing the genetic diversity of their ancestors, it is possible to improve the enzymatic efficiency of RuBisCO.

Glyceraldehyde 3-phosphate dehydrogenase (GAPDH) is a crucial enzyme in the reduction stage of the CBC. According to Petersen et al. (2003), GAPDH plays a significant role in this process. Rius et al. (2006) has reported that if GAPDH is deficient, it can hinder glycolysis and reduce CO_2_ fixation by approximately 25%. The GAPDH is used to create photosynthates and regenerate Ribulose 1,5 bisphosphate. GAPA and GAPB are the two subunits that make up the GAPDH enzyme. Deleting either GAPA or GAPB can significantly reduce carbon assimilation in Arabidopsis. Simkin et al. (2020) have reported that carbon assimilation decreases by 73% by GAPA deletion, while the deletion of GAPB leads to a 34% reduction. In rice, GAPB overexpression increases CO_2_ assimilation and chlorophyll content even under low light conditions (Liu et al., 2020). In contrast, in *Arabidopsis*, carbon assimilation is higher for *gapb* than *gapa*.

Triose phosphate isomerase (TPI) is another essential enzyme in the CBC, playing a pivotal role in the first reaction of the regeneration stage (Johnson, 2016). TPI has a critical C-terminal region, vital for its catalytic, regulatory, or folding function. This region is essential for efficiently converting glyceraldehyde 3-phosphate molecules into dihydroxyacetone phosphate and vice versa (Castro-Torres et al., 2018). Mutations in TPI’s plastid form in *Arabidopsis* result in chlorotic leaves and almost no growth after ten weeks of evaluation because of the accumulation of methylglyoxal, which is twice that of the wild type. As a result, the transition from heterotrophic to autotrophic growth is delayed (Chen & Thelen, 2010). Moreover, TPI has cysteine residues similar to the GAPB subunit of the GAPDH enzyme. In *Arabidopsis* and photosynthetic microorganisms such as *Synechocystis* and *Chlamydomonas*, these residues facilitate its stability and activity by being close to the catalytic site (Dumont et al., 2016; Castro-Torres et al., 2018). In tomato, the mutation of its TPI genes only showed visible phenotype changes in double mutant lines *tpi1tpi2*. In these individuals, Chen et al. (2023) found reduced TPI activity, chlorotic variegation, and reduced carbon-assimilation efficiency in contrast to the wild type. However, assessing the thermostability of TPI1 and TPI2 proteins in tomatoes, the author found that TPI2 may be more stable than TPI1 under heat stress at 42°C.

## 3 The cosmetic and personal care industry: the role of photosynthesis in lycopene production

In addition to ensuring food production, photosynthesis supports plant secondary metabolism since its products are precursors (Qaderi et al., 2023) of over 50 thousand secondary metabolites (Teoh, 2016). Because of their properties, there is growing interest in identifying new secondary metabolites as industry inputs to enhance agricultural sustainability and improve their production (Ozyigit et al., 2023). Thus, plant and food waste are processed as a promise source to obtain secondary metabolite for the cosmetic industry (Faria-Silva et al., 2020).

The cosmetic and personal care industry uses plant-derived secondary metabolites to formulate products (Ribeiro et al., 2015). This market has seen significant growth from 2016 to 2022 (Liyanaarachchi et al., 2018) as consumers prefer natural products (Nadeeshani Dilhara Gamage et al., 2022). Products containing lycopene for skin care are trendy (Choi et al., 2022) due to their antioxidant capacity, improving skin elasticity and hydration (Franco et al., 2021). Thus, lycopene price is over $6000 per kg (Zia-Ul-Haq et al., 2021). Unfortunately, competition with the food industry affects lycopene supplies (Khan et al., 2021). Although lycopene chemical synthesis can be an alternative, the chemical residues in this process affect its overall quality (Li et al., 2022). Therefore, it is relevant to increase its concentration (Costa et al., 2021) to generate a circular production system.

The biosynthesis of lycopene and other carotenoids begins with the 2-C-methyl-D erythritol 4-phosphate pathway that uses glyceraldehyde 3-phosphate (GAP) and pyruvate (Sathasivam et al., 2021) to form 1-deoxy-D-xylulose 5-phosphate (DXP) via 1-deoxy-D-xylulose-5-phosphate synthase (DXS) (Simpson et al., 2016). The manipulation of DXS increases lycopene production (Kang et al., 2005), and its overexpression results in a twofold increase in the carotenoid content (Morris, 2006). The activity of DXS depends on the availability of GAP, GAPDH being the photosynthetic enzyme that generates this molecule (Petersen et al., 2003). GAPDH has predominant activity in photosynthetically active tissues (Kelly and Gibbs, 1973), favoring the biosynthesis of carotenoids such as lutein, beta-carotene, violaxanthin, and neoxanthin (Sun et al., 2018) that protect the photosynthetic apparatus from oxidative photodamage (Kim et al., 2018). In green tissues of plants, the regulation of carotenoid biosynthesis must occur in a coordinated manner with the assembly of the photosynthesis apparatus (Lu and Li, 2008). On the other hand, phytoene synthase (PSY) knock-out, a critical enzyme for carotenoid biosynthesis, completely suppresses photosynthesis (Sun et al., 2018).

## 4 Conclusion

The cosmetic and personal care industry uses plant-derived secondary metabolites, like lycopene, to avert skin photodamage and aging. Therefore, it is necessary to link diversity in plant secondary metabolism with the underlying genetic architecture related to photosynthetic gene diversity (LPE1, HCF173, HCF244, D1, Cytochrome b6f complex and NDH complex subunits, APTase γ subunit, RuBisCO, GAPA, GAPB and TPI) to add value to the Solanaceae biodiversity to develop new crops and thus prevent competition with the food industry.

## Author contributions

CA-B: Writing – original draft, Writing – review & editing. GZ: Conceptualization, Writing – original draft, Writing – review & editing.

## References

[B1] Akiyama K. Ozawa S.-I. Takahashi Y. Yoshida K. Suzuki T. Kondo K.. (2023). Two specific domains of the γ subunit of chloroplast F _o_ F _1_ provide redox regulation of the ATP synthesis through conformational changes. Proc. Natl. Acad. Sci. 120, 1–8. doi: 10.1073/pnas.2218187120 PMC9964038 36716358

[B2] Bhattacharya O. Ortiz I. Hendricks N. Walling L. L. (2023). The tomato chloroplast stromal proteome compendium elucidated by leveraging a plastid protein-localization prediction Atlas. Front. Plant Sci. 14. doi: 10.3389/fpls.2023.1020275 PMC10493611 37701797

[B3] Bollenbach T. J. (2003). Divalent metal-dependent catalysis and cleavage specificity of CSP41, a chloroplast endoribonuclease belonging to the short chain dehydrogenase/reductase superfamily. Nucleic Acids Res. 31, 4317–4325. doi: 10.1093/nar/gkg640 12888490 PMC169913

[B4] Brestic M. Yang X. Li X. Allakhverdiev S. I. (2021). Crop photosynthesis for the twenty-first century. Photosynth Res. 150, 1–3. doi: 10.1007/s11120-021-00869-5 34674135

[B5] Bulut M. Nunes-Nesi A. Fernie A. R. Alseekh S. (2023). Characterization of PetM cytochrome *b6f* subunit 7 domain-containing protein in tomato. Hortic. Res. 10, 1–12. doi: 10.1093/hr/uhad224 PMC10716634 38094587

[B6] Castro-Torres E. Jimenez-Sandoval P. Fernández-de Gortari E. López-Castillo M. Baruch-Torres N. López-Hidalgo M.. (2018). Structural basis for the limited response to oxidative and thiol-conjugating agents by triosephosphate isomerase from the photosynthetic bacteria synechocystis. Front. Mol. Biosci. 5. doi: 10.3389/fmolb.2018.00103 PMC6277545 30538993

[B7] Chen C. Zhang M. Ma X. Meng Q. Zhuang K. (2023). Differential heat-response characteristics of two plastid isoforms of triose phosphate isomerase in tomato. Plant Biotechnol. J. 1–12. doi: 10.1111/pbi.14212 PMC10893939 37878418

[B8] Chen J.-H. Chen S.-T. He N.-Y. Wang Q.-L. Zhao Y. Gao W.. (2020). Nuclear-encoded synthesis of the D1 subunit of photosystem II increases photosynthetic efficiency and crop yield. Nat. Plants 6, 570–580. doi: 10.1038/s41477-020-0629-z 32313138

[B9] Chen M. Thelen J. J. (2010). The plastid isoform of triose phosphate isomerase is required for the postgerminative transition from heterotrophic to autotrophic growth in *Arabidopsis* . Plant Cell 22, 77–90. doi: 10.1105/tpc.109.071837 20097871 PMC2828694

[B10] Cheuk A. Meier T. (2021). Rotor subunits adaptations in ATP synthases from photosynthetic organisms. Biochem. Soc. Trans. 49, 541–550. doi: 10.1042/BST20190936 33890627 PMC8106487

[B11] Choi Y.-H. Kim S. E. Lee K.-H. (2022). Changes in consumers’ awareness and interest in cosmetic products during the pandemic. Fashion Textiles 9, 1. doi: 10.1186/s40691-021-00271-8

[B12] Costa A. Marques M. Congiu F. Paiva A. Simões P. Ferreira A.. (2021). Evaluating the presence of lycopene-enriched extracts from tomato on topical emulsions: physico-chemical characterization and sensory analysis. Appl. Sci. 11, 5120. doi: 10.3390/app11115120

[B13] Demír A. (2021). The impacts of climate change on genetic diversity. Biol. Divers. Conserv. 14/3, 511–518. doi: 10.46309/biodicon.2021.1032772

[B14] Dumont S. Bykova N. V. Pelletier G. Dorion S. Rivoal J. (2016). Cytosolic triosephosphate isomerase from arabidopsis thaliana is reversibly modified by glutathione on cysteines 127 and 218. Front. Plant Sci. 7. doi: 10.3389/fpls.2016.01942 PMC5177656 28066493

[B15] Faria-Silva C. Ascenso A. Costa A. M. Marto J. Carvalheiro M. Ribeiro H. M.. (2020). Feeding the skin: A new trend in food and cosmetics convergence. Trends Food Sci. Technol. 95, 21–32. doi: 10.1016/j.tifs.2019.11.015

[B16] Franco L. Marchena A. Rodríguez A. (2021). Skin health properties of lycopene and melatonin. J. Dermatol. Skin Sci. 3, 26–29. doi: 10.29245/2767-5092/2021/1.1126

[B17] Genkov T. Meyer M. Griffiths H. Spreitzer R. J. (2010). Functional hybrid rubisco enzymes with plant small subunits and algal large subunits. J. Biol. Chem. 285, 19833–19841. doi: 10.1074/jbc.M110.124230 20424165 PMC2888394

[B18] Jin H. Fu M. Duan Z. Duan S. Li M. Dong X.. (2018). LOW PHOTOSYNTHETIC EFFICIENCY 1 is required for light-regulated photosystem II biogenesis in *Arabidopsis* . Proc. Natl. Acad. Sci. 115, E6075–E6084. doi: 10.1073/pnas.1807364115 PMC6042084 29891689

[B19] Johnson M. P. (2016). Photosynthesis. Essays Biochem. 60, 255–273. doi: 10.1042/EBC20160016 27784776 PMC5264509

[B20] Kang M. J. Lee Y. M. Yoon S. H. Kim J. H. Ock S. W. Jung K. H.. (2005). Identification of genes affecting lycopene accumulation in *Escherichia coli* using a shot-gun method. Biotechnol. Bioeng 91, 636–642. doi: 10.1002/bit.20539 15898075

[B21] Kelly G. J. Gibbs M. (1973). Nonreversible d-glyceraldehyde 3-phosphate dehydrogenase of plant tissues. Plant Physiol. 52, 111–118. doi: 10.1104/pp.52.2.111 16658509 PMC366450

[B22] Khan U. M. Sevindik M. Zarrabi A. Nami M. Ozdemir B. Kaplan D. N.. (2021). Lycopene: food sources, biological activities, and human health benefits. Oxid. Med. Cell Longev 2021, 1–10. doi: 10.1155/2021/2713511 PMC8626194 34840666

[B23] Kim H. S. Ji C. Y. Lee C.-J. Kim S.-E. Park S.-C. Kwak S.-S. (2018). Orange: a target gene for regulating carotenoid homeostasis and increasing plant tolerance to environmental stress in marginal lands. J. Exp. Bot. 69, 3393–3400. doi: 10.1093/jxb/ery023 29385615

[B24] Kohzuma K. Dal Bosco C. Kanazawa A. Kramer D. M. Meurer J. (2013). A potential function for the γ2 subunit (atpC2) of the chloroplast ATP synthase. 576–578. Berlin, Heidelberg: Springer. doi: 10.1007/978-3-642-32034-7_123

[B25] Leister D. (2023). Enhancing the light reactions of photosynthesis: Strategies, controversies, and perspectives. Mol. Plant 16, 4–22. doi: 10.1016/J.MOLP.2022.08.005 35996755

[B26] León-Sánchez L. Nicolás E. Nortes P. A. Maestre F. T. Querejeta J. I. (2016). Photosynthesis and growth reduction with warming are driven by nonstomatal limitations in a Mediterranean semi-arid shrub. Ecol. Evol. 6, 2725–2738. doi: 10.1002/ece3.2074 27066247 PMC4798828

[B27] Li M. Xia Q. Lv S. Tong J. Wang Z. Nie Q.. (2022). Enhanced CO _2_ capture for photosynthetic lycopene production in engineered *Rhodopseudomonas palustris*, a purple nonsulfur bacterium. Green Chem. 24, 7500–7518. doi: 10.1039/D2GC02467E

[B28] Lin M. T. Orr D. J. Worrall D. Parry M. A. J. Carmo-Silva E. Hanson M. R. (2021). A procedure to introduce point mutations into the Rubisco large subunit gene in wild-type plants. Plant J. 106, 876–887. doi: 10.1111/tpj.15196 33576096

[B29] Lin M. T. Salihovic H. Clark F. K. Hanson M. R. (2022). Improving the efficiency of Rubisco by resurrecting its ancestors in the family Solanaceae. Sci. Adv. 8, 1–12. doi: 10.1126/sciadv.abm6871 PMC9012466 35427154

[B30] Link S. Engelmann K. Meierhoff K. Westhoff P. (2012). The Atypical Short-Chain Dehydrogenases HCF173 and HCF244 Are Jointly Involved in Translational Initiation of the *psbA* mRNA of *Arabidopsis* . Plant Physiol. 160, 2202–2218. doi: 10.1104/pp.112.205104 23027666 PMC3510141

[B31] Liu Y. Pan T. Tang Y. Zhuang Y. Liu Z. Li P.. (2020). Proteomic analysis of rice subjected to low light stress and overexpression of osGAPB increases the stress tolerance. Rice 13, 30. doi: 10.1186/s12284-020-00390-8 32488648 PMC7266901

[B32] Liyanaarachchi G. D. Samarasekera J. K. R. R. Mahanama K. R. R. Hemalal K. D. P. (2018). Tyrosinase, elastase, hyaluronidase, inhibitory and antioxidant activity of Sri Lankan medicinal plants for novel cosmeceuticals. Ind. Crops Prod 111, 597–605. doi: 10.1016/j.indcrop.2017.11.019

[B33] Lu S. Li L. (2008). Carotenoid metabolism: biosynthesis, regulation, and beyond. J. Integr. Plant Biol. 50, 778–785. doi: 10.1111/j.1744-7909.2008.00708.x 18713388

[B34] Ma M. Liu Y. Bai C. Yong J. W. H. (2021). The significance of chloroplast NAD(P)H dehydrogenase complex and its dependent cyclic electron transport in photosynthesis. Front. Plant Sci. 12. doi: 10.3389/fpls.2021.661863 PMC8102782 33968117

[B35] Malone L. A. Proctor M. S. Hitchcock A. Hunter C. N. Johnson M. P. (2021). Cytochrome b6f – Orchestrator of photosynthetic electron transfer. Biochim. Biophys. Acta (BBA) - Bioenergetics 1862, 148380. doi: 10.1016/j.bbabio.2021.148380 33460588

[B36] Mathur S. Agrawal D. Jajoo A. (2014). Photosynthesis: Response to high temperature stress. J. Photochem. Photobiol. B 137, 116–126. doi: 10.1016/J.JPHOTOBIOL.2014.01.010 24796250

[B37] Mirzabaev A. Bezner Kerr R. Hasegawa T. Pradhan P. Wreford A. Cristina Tirado von der Pahlen M.. (2023). Severe climate change risks to food security and nutrition. Clim Risk Manag 39, 1–10. doi: 10.1016/j.crm.2022.100473

[B38] Monde R. Zito F. Olive J. Wollman F. Stern D. B. (2000). Post-transcriptional defects in tobacco chloroplast mutants lacking the cytochrome *b _6_/f* complex. Plant J. 21, 61–72. doi: 10.1046/j.1365-313x.2000.00653.x 10652151

[B39] Morris W. L. (2006). Overexpression of a bacterial 1-deoxy-D-xylulose 5-phosphate synthase gene in potato tubers perturbs the isoprenoid metabolic network: implications for the control of the tuber life cycle. J. Exp. Bot. 57, 3007–3018. doi: 10.1093/jxb/erl061 16873449

[B40] Muir C. D. Pease J. B. Moyle L. C. (2014). Quantitative genetic analysis indicates natural selection on leaf phenotypes across wild tomato species (*Solanum* sect. *Lycopersicon*; solanaceae). Genetics 198, 1629–1643. doi: 10.1534/genetics.114.169276 25298519 PMC4256776

[B41] Nadeeshani Dilhara Gamage D. G. Dharmadasa R. M. Chandana Abeysinghe D. Saman Wijesekara R. G. Prathapasinghe G. A. Someya T. (2022). Global perspective of plant-based cosmetic industry and possible contribution of Sri Lanka to the development of herbal cosmetics. Evidence-Based Complementary Altern. Med. 2022, 1–26. doi: 10.1155/2022/9940548 PMC8916882 35280508

[B42] Nicolao R. Gaiero P. Castro C. M. Heiden G. (2023). Solanum malmeanum, a promising wild relative for potato breeding. Front. Plant Sci. 13. doi: 10.3389/fpls.2022.1046702 PMC9986444 36891130

[B43] Ozyigit I. I. Dogan I. Hocaoglu-Ozyigit A. Yalcin B. Erdogan A. Yalcin I. E.. (2023). Production of secondary metabolites using tissue culture-based biotechnological applications. Front. Plant Sci. 14. doi: 10.3389/fpls.2023.1132555 PMC10339834 37457343

[B44] Palchetti M. V. Cantero J. J. Barboza G. E. (2020). Solanaceae diversity in South America and its distribution in Argentina. Acad. Bras. Cienc 92, 1–17. doi: 10.1590/0001-3765202020190017 32785441

[B45] Parto S. Lartillot N. (2018). Molecular adaptation in Rubisco: Discriminating between convergent evolution and positive selection using mechanistic and classical codon models. PloS One 13, e0192697. doi: 10.1371/journal.pone.0192697 29432438 PMC5809049

[B46] Peng W. Berry E. M. (2018). “The concept of food security,” in Encyclopedia of food security and sustainability (Elsevier), 1–7. doi: 10.1016/B978-0-08-100596-5.22314-7

[B47] Petersen J. Brinkmann H. Cerff R. (2003). Origin, evolution, and metabolic role of a novel glycolytic GAPDH enzyme recruited by land plant plastids. J. Mol. Evol. 57, 16–26. doi: 10.1007/s00239-002-2441-y 12962302

[B48] Qaderi M. M. Martel A. B. Strugnell C. A. (2023). Environmental factors regulate plant secondary metabolites. Plants 12, 447. doi: 10.3390/plants12030447 36771531 PMC9920071

[B49] Ribeiro A. Estanqueiro M. Oliveira M. Sousa Lobo J. (2015). Main benefits and applicability of plant extracts in skin care products. Cosmetics 2, 48–65. doi: 10.3390/cosmetics2020048

[B50] Rius S. P. Casati P. Iglesias A. A. Gomez-Casati D. F. (2006). Characterization of an Arabidopsis thaliana mutant lacking a cytosolic non-phosphorylating glyceraldehyde-3-phosphate dehydrogenase. Plant Mol. Biol. 61, 945–957. doi: 10.1007/s11103-006-0060-5 16927206

[B51] Rochaix J.-D. (2011). Reprint of: Regulation of photosynthetic electron transport. Biochim. Biophys. Acta (BBA) - Bioenergetics 1807, 878–886. doi: 10.1016/j.bbabio.2011.05.009 21605544

[B52] Rott M. Martins N. F. Thiele W. Lein W. Bock R. Kramer D. M.. (2011). ATP synthase repression in tobacco restricts photosynthetic electron transport, CO _2_ assimilation, and plant growth by overacidification of the thylakoid lumen. Plant Cell 23, 304–321. doi: 10.1105/tpc.110.079111 21278125 PMC3051256

[B53] Salgotra R. K. Chauhan B. S. (2023). Genetic diversity, conservation, and utilization of plant genetic resources. Genes (Basel) 14, 174. doi: 10.3390/genes14010174 36672915 PMC9859222

[B54] Samuels J. (2015). Biodiversity of food species of the solanaceae family: A preliminary taxonomic inventory of subfamily solanoideae. Resources 4, 277–322. doi: 10.3390/resources4020277

[B55] Sathasivam R. Radhakrishnan R. Kim J. K. Park S. U. (2021). An update on biosynthesis and regulation of carotenoids in plants. South Afr. J. Bot. 140, 290–302. doi: 10.1016/j.sajb.2020.05.015

[B56] Schult K. Meierhoff K. Paradies S. Toöller T. Wolff P. Westhoff P. (2007). The Nuclear-Encoded Factor HCF173 Is Involved in the Initiation of Translation of the *psbA* mRNA in *Arabidopsis thaliana* . Plant Cell 19, 1329–1346. doi: 10.1105/tpc.106.042895 17435084 PMC1913763

[B57] Sello S. Meneghesso A. Alboresi A. Baldan B. Morosinotto T. (2019). Plant biodiversity and regulation of photosynthesis in the natural environment. Planta 249, 1217–1228. doi: 10.1007/s00425-018-03077-z 30607502

[B58] Shimizu H. Peng L. Myouga F. Motohashi R. Shinozaki K. Shikanai T. (2008). CRR23/ndhL is a subunit of the chloroplast NAD(P)H dehydrogenase complex in Arabidopsis. Plant Cell Physiol. 49, 835–842. doi: 10.1093/pcp/pcn058 18388109

[B59] Simkin A. J. Faralli M. Ramamoorthy S. Lawson T. (2020). Photosynthesis in non-foliar tissues: implications for yield. Plant J. 101, 1001–1015. doi: 10.1111/tpj.14633 31802560 PMC7064926

[B60] Simpson K. Quiroz L. F. Rodriguez-Concepción M. Stange C. R. (2016). Differential contribution of the first two enzymes of the MEP pathway to the supply of metabolic precursors for carotenoid and chlorophyll biosynthesis in carrot (Daucus carota). Front. Plant Sci. 7. doi: 10.3389/fpls.2016.01344 PMC5005961 27630663

[B61] Sirpiö S. Allahverdiyeva Y. Holmström M. Khrouchtchova A. Haldrup A. Battchikova N.. (2009). Novel nuclear-encoded subunits of the chloroplast NAD(P)H dehydrogenase complex. J. Biol. Chem. 284, 905–912. doi: 10.1074/JBC.M805404200 18974055

[B62] Sukenik A. Bennett J. Falkowski P. (1987). Light-saturated photosynthesis — Limitation by electron transport or carbon fixation? Biochim. Biophys. Acta (BBA) - Bioenerg. 891, 205–215. doi: 10.1016/0005-2728(87)90216-7

[B63] Sun T. Yuan H. Cao H. Yazdani M. Tadmor Y. Li L. (2018). Carotenoid metabolism in plants: the role of plastids. Mol. Plant 11, 58–74. doi: 10.1016/j.molp.2017.09.010 28958604

[B64] Teoh E. S. (2016). “Secondary metabolites of plants,” in Medicinal orchids of asia (Springer International Publishing, Cham), 59–73. doi: 10.1007/978-3-319-24274-3_5

[B65] van Bel A. J. E. Offler C. E. Patrick J. W. (2003). “PHOTOSYNTHESIS AND PARTITIONING | Sources and sinks,” in Encyclopedia of applied plant sciences (Elsevier), 724–734. doi: 10.1016/B0-12-227050-9/00089-2

[B66] Wang P. Duan W. Takabayashi A. Endo T. Shikanai T. Ye J.-Y.. (2006). Chloroplastic NAD(P)H dehydrogenase in tobacco leaves functions in alleviation of oxidative damage caused by temperature stress. Plant Physiol. 141, 465–474. doi: 10.1104/pp.105.070490 16428601 PMC1475475

[B67] Wang Z. Li G. Sun H. Ma L. Guo Y. Zhao Z.. (2018). Effects of drought stress on photosynthesis and photosynthetic electron transport chain in young apple tree leaves. Biol. Open. 7, 1–9. doi: 10.1242/bio.035279 PMC6262865 30127094

[B68] World Economic Forum. (2023). The Global Risks Report 2023 18th Edition. Geneva.

[B69] Yadav S. K. Khatri K. Rathore M. S. Jha B. (2018). Introgression of UfCyt c6, a thylakoid lumen protein from a green seaweed Ulva fasciata Delile enhanced photosynthesis and growth in tobacco. Mol. Biol. Rep. 45, 1745–1758. doi: 10.1007/s11033-018-4318-1 30159639

[B70] Yadav S. K. Khatri K. Rathore M. S. Jha B. (2020). Ectopic Expression of a Transmembrane Protein KaCyt b _6_ from a Red Seaweed *Kappaphycus alvarezii* in Transgenic Tobacco Augmented the Photosynthesis and Growth. DNA Cell Biol. 1–15. doi: 10.1089/dna.2020.5479 32865429

[B71] Zhang L. Paakkarinen V. van Wijk K. J. Aro E.-M. (2000). Biogenesis of the chloroplast-encoded D1 protein: regulation of translation elongation, insertion, and assembly into photosystem II. Plant Cell 12, 1769–1781. doi: 10.1105/tpc.12.9.1769 11006346 PMC149084

[B72] Zhao C. Liu B. Piao S. Wang X. Lobell D. B. Huang Y.. (2017). Temperature increase reduces global yields of major crops in four independent estimates. Proc. Natl. Acad. Sci. 114, 9326–9331. doi: 10.1073/pnas.1701762114 PMC5584412 28811375

[B73] Zhao H. Tang Q. Chang T. Xiao Y. Zhu X.-G. (2021). Why an increase in activity of an enzyme in the Calvin–Benson cycle does not always lead to an increased photosynthetic CO2 uptake rate?—a theoretical analysis. In Silico Plants 3, 1–13. doi: 10.1093/insilicoplants/diaa009

[B74] Zhu X.-G. Long S. P. Ort D. R. (2010). Improving photosynthetic efficiency for greater yield. Annu. Rev. Plant Biol. 61, 235–261. doi: 10.1146/annurev-arplant-042809-112206 20192734

[B75] Zia-Ul-Haq M. Dewanjee S. Riaz M. (2021). Carotenoids: structure and function in the human body (Cham: Springer International Publishing). doi: 10.1007/978-3-030-46459-2

